# Case Report: Micafungin for treating *Candida glabrata* urinary infection: a clinical case in a premature neonate

**DOI:** 10.3389/fped.2024.1397456

**Published:** 2024-05-17

**Authors:** Carlos Javier Parramon-Teixido, Carme Garcia Esquerda, Marie Antoinette Frick, Cinzia Tripodi, Laura Gomez-Ganda, Cesar Wenceslao Ruiz-Campillo, Maria Josep Cabañas-Poy

**Affiliations:** ^1^ Department of Pharmacy, Vall d’Hebron University Hospital, Barcelona, Spain; ^2^ Division of Infectious Diseases and Pediatric Immunology, Department of Pediatrics, Vall d’Hebron University Hospital, Barcelona, Spain; ^3^ Department of Neonatology, Dr. Josep Trueta University Hospital of Girona, Girona, Spain; ^4^ Department of Neonatology, Vall d’Hebron University Hospital, Barcelona, Spain

**Keywords:** *Candida glabrata*, micafungin, urinary tract infection, premature infant, case report

## Abstract

Urinary tract infections (UTIs) associated with indwelling urinary catheterization (IUC) in premature newborns (PNBs) pose a significant challenge in neonatal intensive care units (NICUs) due to the vulnerability of this population to infections and the necessity of invasive procedures. While bacterial UTIs have historically been predominant, there is a rising incidence of fungal pathogens, particularly non-*albicans Candida* strains like *Candida glabrata* and *Candida tropicalis*, attributed to broad-spectrum antibiotic use. Diagnosis of fungal UTIs in a PNB relies on culturing *Candida* spp. from properly collected urine samples, particularly critical in very low birth weight (VLBW) PNBs because of the risk of invasive candidiasis and associated complications. We present a case of an extremely premature newborn (EPNB) successfully treated for a UTI caused by *C. glabrata* with micafungin. Our case exhibits micafungin as a potentially safe and effective alternative for treating *C. glabrata* UTIs in neonates.

## Introduction

Urinary tract infections (UTIs) associated with indwelling urinary catheterization (IUC) (often required to ensure continuous drainage and measures of urinary output) in premature newborns (PNBs) represent a nosocomial infection prevalent within neonatal intensive care units (NICUs), owing to the increased susceptibility to infections in this population and the required employment of invasive procedures and supportive devices (1). Although UTIs are predominantly bacterial, the incidence of fungal pathogens in UTIs is on the rise (2, 3). Despite *Candida albicans* being the predominant pathogenic species historically, recent years have witnessed a notable surge in UTIs attributed to non-*albicans Candida* strains (30%–50%), notably *Candida glabrata* and *Candida tropicalis*, attributable to the widespread use of broad-spectrum antibiotics (3).

The acquisition of a positive *Candida* spp. culture (obtained through sterile technique) from urine in the absence of candidemia is pivotal in diagnosing UTIs caused by fungal pathogen (4). Candiduria in very low birth weight (VLBW) PNBs is associated with a significant risk of invasive candidiasis, which can lead to central nervous system involvement and mortality (4, 5). Symptoms of candidiasis in PNBs are more non-specific compared to other populations, posing challenges in diagnosis, thus necessitating comprehensive evaluation and early initiation of antifungal therapy (4).

A definitive diagnosis of UTIs in a PNB relies on culturing any microorganism from a properly collected urine sample before treatment initiation. To obtain a urine culture, the gold standard technique is suprapubic aspiration (SPA); alternatively, sterilely collected urine via catheterization (CATH) is considered. Significant bacteriuria is defined as any bacterial count in urine obtained by SPA or >10,000 colony-forming units (CFU)/ml in samples collected by CATH (5).

In the event of a suspected UTI in a PNB, if feasible, the urinary catheter should be replaced to exclude potential colonization. Treatment of fungal UTIs is recommended solely for patients at high risk of dissemination, such as VLBW PNBs, who should be managed as though they have candidemia (4). Antifungal agents are selected based on the sensitivity of the infecting species and their ability to achieve adequate concentrations in both blood and urine (4–6). Fluconazole is the preferred treatment due to its favorable urinary excretion, enabling sufficient concentrations in urine (2, 4). Nonetheless, the rise in non-*albicans Candida* UTIs has prompted changes in resistance profiles. *C. glabrata* demonstrates dose-dependent resistance to fluconazole, while *Candida krusei* is inherently resistant (2). Other azoles (voriconazole, itraconazole, and posaconazole) are deemed less efficacious owing to their limited urinary excretion (4).

Although amphotericin B (AMB) deoxycholate has been recommended as the first-line treatment for UTI in guidelines, currently, it is no longer used in clinical practice due to its significant renal toxicity, rendering it unsuitable for PNBs (2, 4, 6). The administration of liposomal AMB is discouraged for UTI owing to its reduced renal penetration (4, 7).

These circumstances have presented a therapeutic challenge in the treatment of non-*albicans Candida* candiduria, prompting exploration of alternative options such as echinocandins. Echinocandins, through their mechanism of β-glucan synthase inhibition, exhibit fungicidal activity against *Candida* spp. (3, 4). Furthermore, they demonstrate a favorable adverse effect and drug interaction profile compared to azoles (3, 4, 8). However, their urinary excretion percentages in their active form are notably low: 1.4%, 0.7%, and 0.1% for caspofungin, micafungin, and anidulafungin, respectively, limiting their utility in UTI management (9, 10). In terms of pharmacokinetics, micafungin is rapidly and moderately distributed to renal tissues and the bladder, likely explaining its efficacy in these infections (10). In addition, it attains high plasma concentrations, which, despite its low urinary excretion, may yield sufficiently elevated levels in urine to achieve an optimal maximum concentration (Cmax) to minimum inhibitory concentration (MIC) (Cmax/MIC) ratio for UTI treatment (10).

Although recent studies have reported successful micafungin treatment of *Candida* spp. UTIs in hospitalized adults and pediatric patients (3, 8–10), there are currently no data regarding its application in PNBs. Therefore, we present a case of an extremely premature newborn (EPNB) effectively treated for fungal UTI with micafungin.

## Case description

**Figure 1:**
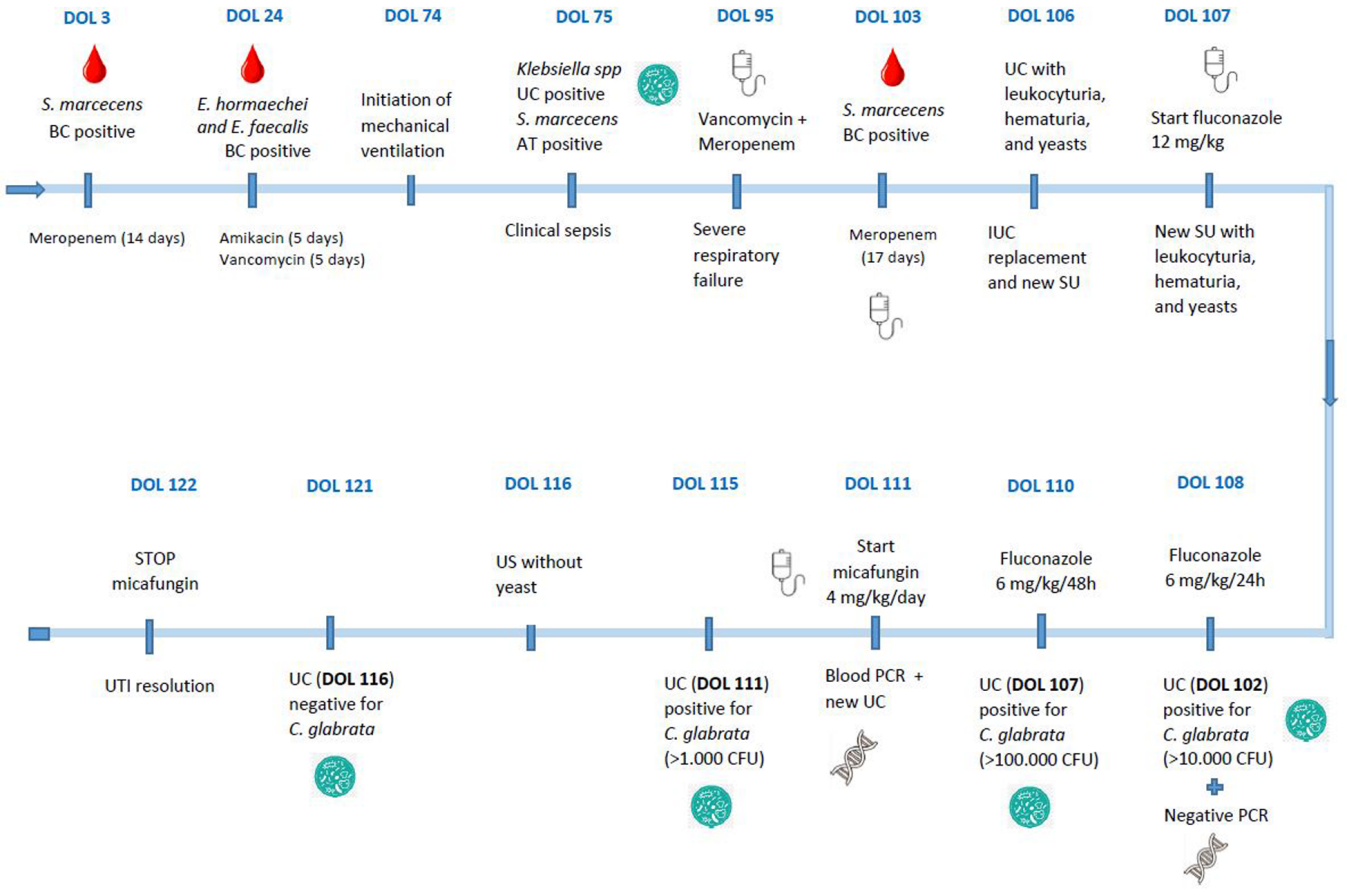
Sequence of events of the patient. BC, blood culture; CFU, colony-forming unit; DOL, day of life; IUC, indwelling urinary catheter; UC, urine culture; US, urine sediment; UTI, urinary tract infection.

A female EPNB was born at 26 weeks of gestation with a birth weight of 709 g. Notably, severe bronchopulmonary dysplasia with secondary pulmonary hypertension was observed during the first months of life, requiring mechanical ventilation since day 74 of life (DOL). The patient presented three episodes of nosocomial sepsis within the first 3 months. The first episode was at 3 DOL with a positive blood culture for *Serratia marcescens*, the second episode at 24 DOL with a positive blood culture for *Enterobacter hormaechei* and *Enterococcus faecalis*, and the last one at 75 DOL with positive urine culture for *Klebsiella* spp. and tracheal aspiration (TA) for *S. marcescen*s (Figure 1). The patient has received various antibiotics (amikacin for 5 days, vancomycin for 5 days, and meropenem for 14 and 17 days).

At 95 DOL, the patient was transferred to our center due to severe respiratory failure. Given her critical condition, a clinical and laboratory suspicion of sepsis, and a positive TA for *S. marcescens*, meropenem and vancomycin were initiated. Blood cultures, urine cultures (UC), TA, and urine sediment (US) (patient with IUC) were performed, and the previous epicutaneous catheter was removed. The patient's IUC was not removed because it was essential for monitoring urinary output. At 103 DOL, the blood culture yielded a positive result for *S. marcescens*, leading to the discontinuation of vancomycin and continuation of meropenem treatment for 14 days.

Leukocyturia, hematuria, and abundant yeasts were observed in the US. Consequently, the IUC was replaced, and a second US showed the presence of yeasts. A polymerase chain reaction (PCR) for *Candida* spp. in blood was performed, and treatment with fluconazole was initiated at a loading dose of 12 mg/kg followed by 6 mg/kg/day, as per the usual practice of our center. Three days later, the urine culture yielded a positive result for *C. glabrata* (>10,000 CFU), with a negative blood PCR. Fluconazole treatment was continued, and an invasive fungal infection workup was negative.

After 4 days of fluconazole treatment, and after the replacement of the IUC, a new urine culture showed persistently positive results for *C. glabrata* (>100,000 CFU). An antibiogram revealed intermediate sensitivity of *C. glabrata* to fluconazole (MIC: 8 µg/ml) and sensitivity to echinocandins (caspofungin: MIC 0.06 µg/ml; anidulafungin: MIC 0.03 µg/ml; micafungin: MIC 0.015 µg/ml). Given the presence of two positive urine cultures for *C. glabrata*, along with a new elevation of acute-phase reactants and no clear clinical changes, a multidisciplinary meeting was held with the NICU medical team, the Pediatric Infectious Diseases Unit, and the Pharmacy Department. A literature search for possible therapeutic alternatives suggested the use of echinocandins, leading to the discontinuation of fluconazole and initiation of micafungin treatment. Before starting the new treatment, a new *Candida* spp. study in urine was conducted, which still showed positive results for *C. glabrata* (>10,000 CFU), with a negative blood PCR.

Micafungin treatment was initiated at a dose of 4 mg/kg/day. After 5 days of treatment, a urine culture with US showed the absence of yeasts. Based on these findings, it was decided to complete 14 days of micafungin at the same dose, while awaiting the urine culture result. Finally, the urine culture showed a negative result for *C. glabrata*, and clinical improvement was observed, so it was decided to discontinue antibiotic therapy, concluding the eradication of the UTI. No clinical or analytical adverse effects associated with micafungin were reported during or in the days after treatment. The patient died a week later due to pulmonary complications arising from her underlying lung pathology.

## Discussion

To the best of our knowledge, we present the first reported clinical case in the literature of an EPNB with VLBW successfully treated for a UTI caused by *C. glabrata* using micafungin.

The dosing of micafungin in neonates for candidemia according to NeoFax recommendations is 4 mg/kg/day (11), as was administered in the present case. However, for the treatment of candiduria, dosing is not firmly established. Recently, Ekinci et al. (3) conducted the first retrospective cohort study in a pediatric population, where 24 patients with fungal UTIs were treated with micafungin at a dose of 2 mg/kg/day, yielding satisfactory results and only one adverse event reported (transaminase increased in one patient). It is noteworthy that in this study, the age of the youngest patient was 2 months (3), an age at which the 2 mg/kg/day dose of micafungin is appropriate (12).

On the other hand, evidence of micafungin use for treating fungal UTIs caused by various *Candida* spp. in adults is more extensive, with several publications covering hundreds of patients (3, 4, 8–10, 13). In terms of the safety profile of micafungin utilization, its propensity for inducing alterations in hepatic biomarkers is widely acknowledged (12). In this context, Auriti et al. (14) investigated the administration of micafungin at elevated doses (8–15 mg/kg/day) in neonates afflicted with systemic candidiasis, observing an elevation in hepatic enzymes, namely alkaline phosphatase and gamma-glutamyltransferase. In that case, no discernible analytical alterations were noted throughout the treatment or in the immediate aftermath of its cessation.

Given that we present a single clinical case and that there may be considerable variability in patients’ evolution, especially EPNBs with VLBW, we stress the necessity for further studies and clinical trials in this population, where the available evidence is limited.

Therefore, we conclude that the administration of micafungin in neonates with UTI caused by *C. glabrata* appears to be a safe and effective alternative that could facilitate yeast eradication and patient recovery.

## Data Availability

The original contributions presented in the study are included in the article/Supplementary Material, further inquiries can be directed to the corresponding author.
